# Comparative Analysis of Matrix Metalloproteinase Family Members Reveals That *MMP9* Predicts Survival and Response to Temozolomide in Patients with Primary Glioblastoma

**DOI:** 10.1371/journal.pone.0151815

**Published:** 2016-03-29

**Authors:** Qingbin Li, Baoshi Chen, Jinquan Cai, Ying Sun, Guangzhi Wang, Yongli Li, Ruiyan Li, Yan Feng, Bo Han, Jianlong Li, Yu Tian, Liye Yi, Chuanlu Jiang

**Affiliations:** 1 Department of Neurosurgery, The Second Affiliated Hospital of Harbin Medical University, Harbin, China; 2 Department of Neurosurgery, Beijing Tiantan Hospital, Capital Medical University, Beijing, China; 3 Chinese Glioma Cooperative Group (CGCG), Beijing, China; University of Portsmouth, School of Pharmacy & Biomedical Sciences, UNITED KINGDOM

## Abstract

**Background:**

Glioblastoma multiform (GBM) is the most common malignant primary brain tumor in adults. Radiotherapy plus concomitant and adjuvant TMZ chemotherapy is the current standard of care for patients with GBM. Matrix metalloproteinases (MMPs), a family of zinc-dependent endopeptidases, are key modulators of tumor invasion and metastasis due to their ECM degradation capacity. The aim of the present study was to identify the most informative MMP member in terms of prognostic and predictive ability for patients with primary GBM.

**Method:**

The mRNA expression profiles of all MMP genes were obtained from the Chinese Glioma Genome Atlas (CGGA), the Repository for Molecular Brain Neoplasia Data (REMBRANDT) and the GSE16011 dataset. MGMT methylation status was also examined by pyrosequencing. The correlation of *MMP9* expression with tumor progression was explored in glioma specimens of all grades. Kaplan–Meier analysis and Cox proportional hazards regression models were used to investigate the association of *MMP9* expression with survival and response to temozolomide.

**Results:**

*MMP9* was the only significant prognostic factor in three datasets for primary glioblastoma patients. Our results indicated that *MMP9* expression is correlated with glioma grade (p<0.0001). Additionally, low expression of M*MP9* was correlated with better survival outcome (OS: p = 0.0012 and PFS: p = 0.0066), and MMP9 was an independent prognostic factor in primary GBM (OS: p = 0.027 and PFS: p = 0.032). Additionally, the GBM patients with low *MMP9* expression benefited from temozolomide (TMZ) chemotherapy regardless of the *MGMT* methylation status.

**Conclusions:**

Patients with primary GBMs with low *MMP9* expression may have longer survival and may benefit from temozolomide chemotherapy.

## Introduction

Glioblastoma multiform (GBM) is the most common malignant primary brain tumor, accounting for 15.6% of all primary brain tumors and 45.2% of primary malignant brain tumors[1]. The 5-year survival rate of GBM patients is less than 5% [2]. Such suboptimal efficacy in primary GBM management is partially attributed to the highly invasive nature of glioma cells, which are capable of diffusely infiltrating and widely migrating into the surrounding brain tissue[3]. Furthermore, invasive tumor cells can escape surgical removal and are relatively resistant to radiation therapy and chemotherapy[4]. Due to the unsatisfactory efficacy of the current treatments for primary GBM, there is an unmet medical need for clinical biomarkers that can predict patient survival and response to treatment.

Recent studies focusing on the mechanisms of glioma invasion suggested a role of matrix metalloproteinases (MMPs) in the process of glioma cell invasion [3]. MMPs, a family of zinc-dependent endopeptidases[5], regulate tumor invasion and metastasis through their extracellular matrix (ECM) degradation capacity in the extracellular milieu of various tissues[6–10]. Although MMP expression levels are highly variable from one tumor to another[11, 12], their increased expression suggests a close association with malignant progression of various human cancers[13–16]. Mounting evidence has demonstrated that increased MMPs expression is related to poor prognosis in the majority of human tumors, including glioma[17–21]. Many studies have confirmed the association between the expression of *MMP1*, *2*, *7*, *9*, *11*, *12*, *14*, *15*, *25* and the tumor grade, whereas that *MMP3*, *8*, *10*, *13*, *16*, *17*, *20*, *21*, *23*, *26*, *27* and *28* do not seem to play a major role in glioblastoma development[22–28]. The available data for *MMP19* and *24* are contradictory, as some studies suggest their involvement during the development of astrocytic tumors[12, 29], and while others do not[30].

**Fig 1:**
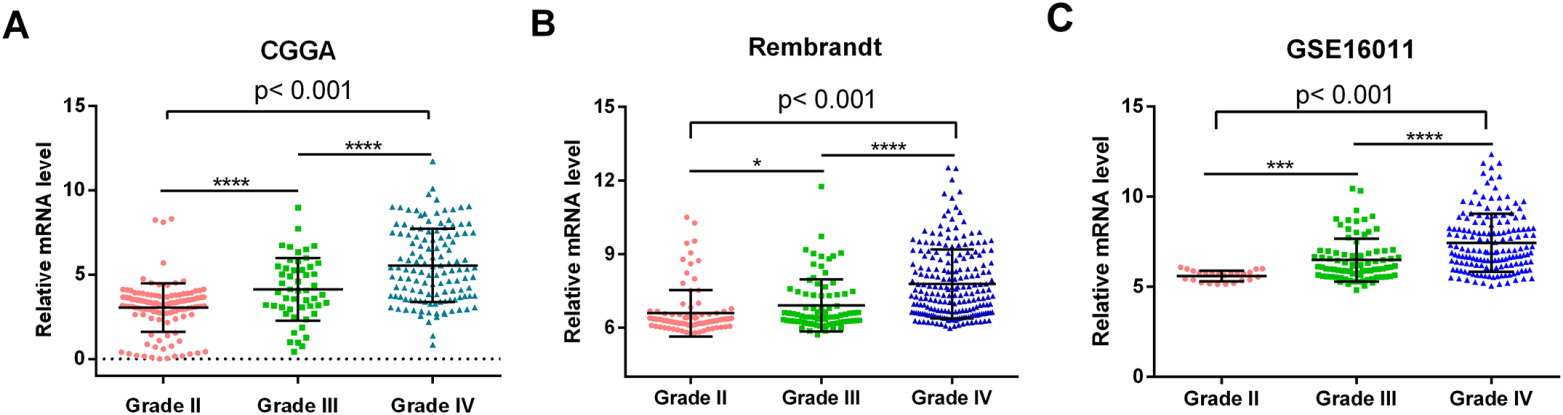
*MMP9* expression was correlated with glioma grade. (A) *MMP9* expression was correlated with glioma grade (p<0.001). Glioma of grade IV showed a significantly increased in *MMP9* expression compared to grade II and III gliomas (p<0.0001, p<0.0001, respectively). *MMP9* expression level in grade III gliomas was markedly higher than that in grade II gliomas (p<0.0001). (B, C) Likelihood ratio test showed that *MMP9* was significantly associated with tumor grade in two independent glioma dataset (p<0.001, p<0.001, respectively) * p< 0.05; **** p<0.0001.

In the present study, we comparatively analyzed the MMP family members based on whole-gene expression profiling from multiple databases (Table 1), and found that *MMP9* expression is correlated with glioma grade (p<0.0001, Fig 1A) and that low *MMP9* expression is an independent prognostic factor for better survival in primary GBM patients (OS: p = 0.027 and PFS: p = 0.032). In addition, low MMP*9* expression was found to be associated with a good response to temozolomide therapy among other clinicopathologic factors. It may contribute to the reasonable usage of TMZ.

**Table 1:** The associations of MMPs with overall survival (OS).

MMPs	HR	95%CI	p value
***MMP9***	1.2048	1.0889–1.3331	0.0003
***MMP1***	1.1671	1.055–1.291	0.0027
***MMP19***	1.2371	1.0463–1.4627	0.0128
***MMP7***	1.1012	1.0133–1.1967	0.0231
***MMP28***	0.6625	0.4441–0.9883	0.0436
***MMP11***	1.1354	1.0006–1.2883	0.0489
***MMP22***	1.2494	0.9855–1.5839	0.0659
***MMP12***	1.1244	0.9909–1.2759	0.0691
***MMP24***	0.7787	0.5731–1.0579	0.1096
***MMP14***	1.1153	0.947–1.3134	0.191
***MMP10***	1.1044	0.9286–1.3135	0.2616
***MMP13***	1.0672	0.9492–1.1998	0.2765
***MMP3***	1.0746	0.9313–1.24	0.3243
***MMP25***	0.8812	0.6678–1.1627	0.3712
***MMP16***	0.9066	0.7282–1.1287	0.3804
***MMP21***	0.8585	0.5884–1.2524	0.4283
***MMP17***	0.8961	0.6669–1.2041	0.4666
***MMP8***	1.0559	0.8497–1.3122	0.6237
***MMP2***	0.9599	0.784–1.1753	0.6918
***MMP20***	0.9628	0.7784–1.1909	0.727
***MMP26***	0.9722	0.7757–1.2184	0.8065
***MMP15***	1.0232	0.7209–1.4523	0.8977
***MMP27***	1.0045	0.8119–1.2426	0.9674

Abbreviations: MMP: Matrix metalloproteinase; HR: hazard ratio.

## Materials and Methods

### Datasets used in this study

Whole genome mRNA expression microarray data and clinical information of 305 glioma and five normal brain samples from the Chinese Glioma Genome Atlas (CGGA) database[31] (http://www.cgga.org.cn) were obtained as a testing set, and this dataset contains 126 grade II, 51 grade III and 128 grade IV samples histologically diagnosed according to the 2007 World Health Organization classification of tumors of the central nervous system[32]. Seventy-eight primary GBM samples with complete clinical information were included in prognostic analysis. These 78 patients underwent surgical resection and then received standard radiation therapy (RT). Fifty of them received adjuvant temozolomide (TMZ) chemotherapy. Written informed consent was obtained from the patients for the publication of this report. The study was performed with the approval of Ethics Committee of Capital Medical University and Harbin Medical University in compliance with the Helsinki Declaration. We also obtained Gene Expression Profiles of two public datasets as our study validation sets including the Repository for Molecular Brain Neoplasia Data (REMBRANDT, n = 433) and the GSE16011 dataset[33] (n = 272). The three datasets were designed as retrospective studies [33–35] providing stable and basic tools for glioma research. They are very mature and suitable for glioma investigation, used widely in several teams of glioma research [33–39]. GSE16011 dataset [33] only has one batch (http://www.ncbi.nlm.nih.gov/geo/query/acc.cgi?acc=GSE16011). Genechips with a glyceraldehyde-3-phosphate dehydrogenase 5′/3′ ratio >4, present calls <30%, unsuccessful RT controls, or a background >200 were excluded. Robustness of sample processing was assessed using eight biological replicates and three technical replicates. Replicates were not included in any analysis. Rembrandt [34] contains data generated through the Glioma Molecular Diagnostic Initiative from glioma specimens comprising gene expression arrays, copy number arrays and clinical phenotype data. Data can be queried and visualized for a selected gene across all data platforms or for multiple genes in a selected platform (https://wiki.nci.nih.gov/display/caIntegrator/caIntegrator+Directory). The CGGA gene expression profile included two batches. These two batches were both detected by the same array- the Agilent Whole Human Genome Array and the data was normalized. The detailed description was illustrated by the Yan et al’s paper [35]. Although batch effects can be reduced by careful experimental design, they cannot be eliminated unless the whole study is done in a single batch. Thus, the data have been computationally corrected using methods such as Bayes [40–42]. S1 Table has illustrated the basic information of the CGGA dataset and the two independent datasets.

### Pyrosequencing for *IDH1* Mutation and *MGMT* Promoter Methylation

Genomic DNA was isolated from frozen tissues with a QIAamp DNA Mini Kit (Qiagen) following the manufacturer’s protocol. DNA concentration and quality were evaluated with a Nano-Drop ND-1000 spectrophotometer (NanoDrop Technologies, Houston, TX). Pyrosequencing for isocitrate dehydrogenase 1 (*IDH1*) mutations[43] and O-6-methylguanine-DNA methyltransferase (*MGMT*) promoter methylation was performed using the PyroMark Q96 ID System (Qiagen, Valencia, Calif)[44]. For *IDH1* mutation, the primers 5'-GCTTGTGAGTGGATGGGTAAAAC-3' and 5'-biotin-TTGCCAACATGACTTACTTGATC-3' were used for PCR amplification, and the primer 5-TGGATGGGTAAAACCT-3' was used for pyrosequencing. For *MGMT* promoter methylation, bisulfite modification of the DNA was performed using the EpiTect Kit (Qiagen); the primers 5'-GTTTYGGATATGTTGG GATA-3' and 5'-biotin-ACCCAAACACTCACCAAATC-3' were used for PCR, and the primer 5'-GGATATGTTGGGATAGT-3' was used for pyrosequencing.

### Statistical Analysis

The prognostic value of all MMP family genes with regards to patient survival was calculated by the Kaplan–Meier method with the two-sided log-rank test (survival) of R, which is an open source statistical software (https://www.r-project.org/). The permuted p-value for each gene was corrected by multiple comparison correction using the Benjamini–Hochberg false discovery rate (FDR). Likelihood ratio test was used to test for differences between at least three groups. Differences in clinicopathologic characteristics between the low and high *MMP9* expression groups (designated using the median level of *MMP9* expression as the cutoff value) were evaluated using the chi-square test. Kaplan-Meier survival analysis was used to estimate the survival distributions. The log-rank test in GraphPad Prism, version 4.0 statistical software was used to assess the statistical significance between stratified survival groups. Cox proportional hazard regression analyses were performed using SPSS, version 19.0, software for Windows (SPSS). For all data, the significance level was set at p < 0.05.

## Results

### *MMP9* was identified as a prognostic biomarker of primary glioblastoma among MMPs in multiple datasets

Firstly, the prognostic value of all genes in the MMP family genes in regards to patient survival were calculated for 78 patients with primary GBM from the CGGA dataset. The following MMP members had prognostic value: *MMP9*, *MMP1*, *MMP19*, *MMP7*, *MMP28*, *and MMP11* (Table 1). In addition, we performed multivariate Cox analysis for the MMP members with significant prognostic value in univariate Cox analysis, only the prognostic values of *MMP9* and *MMP11* remained significant. (*MMP9*: S2 Table; HR, 1.395; 95%CI, 1.144–1.701; p = 0.001). Although the p value of *MMP11* indicated that it had significant prognostic value, its HR value was not stable (0.76 in the multivariate cox regression analysis and 1.1354 in the univariate cox regression analysis). Then, we investigated the public datasets GSE16011 and Rembrandt and found that *MMP9* was the only MMP that could be confirmed to be associated with survival (S3 Table). These results indicated that *MMP9* was a significant prognostic factor among the MMPs.

### Correlation of *MMP9* mRNA expression with grade progression

The expression level of *MMP9* was analyzed in different grades of glioma (Grade II, n = 126; Grade III, n = 51; and Grade IV, n = 128). *MMP9* expression was correlated with grade progression (p<0.001, Fig 1A). As shown in Fig 1A, *MMP9* expression was significantly increased in grade IV glioma compared to in grade II and III gliomas (p<0.0001 and p<0.0001, respectively) and was also markedly higher in grade III glioma than in grade II glioma (p<0.0001). Next, we employed two independent glioma gene expression dataset (REMBRANDT and GSE16011 datasets) to examine the association between *MMP9* expression level and glioma grade. The results showed that *MMP9* was significantly associated with tumor grade (p<0.001, Fig 1B; p<0.001, Fig 1C), which was consistent with our results.

### *MMP9* is an independent prognostic factor in primary GBM patients

**Fig 2:**
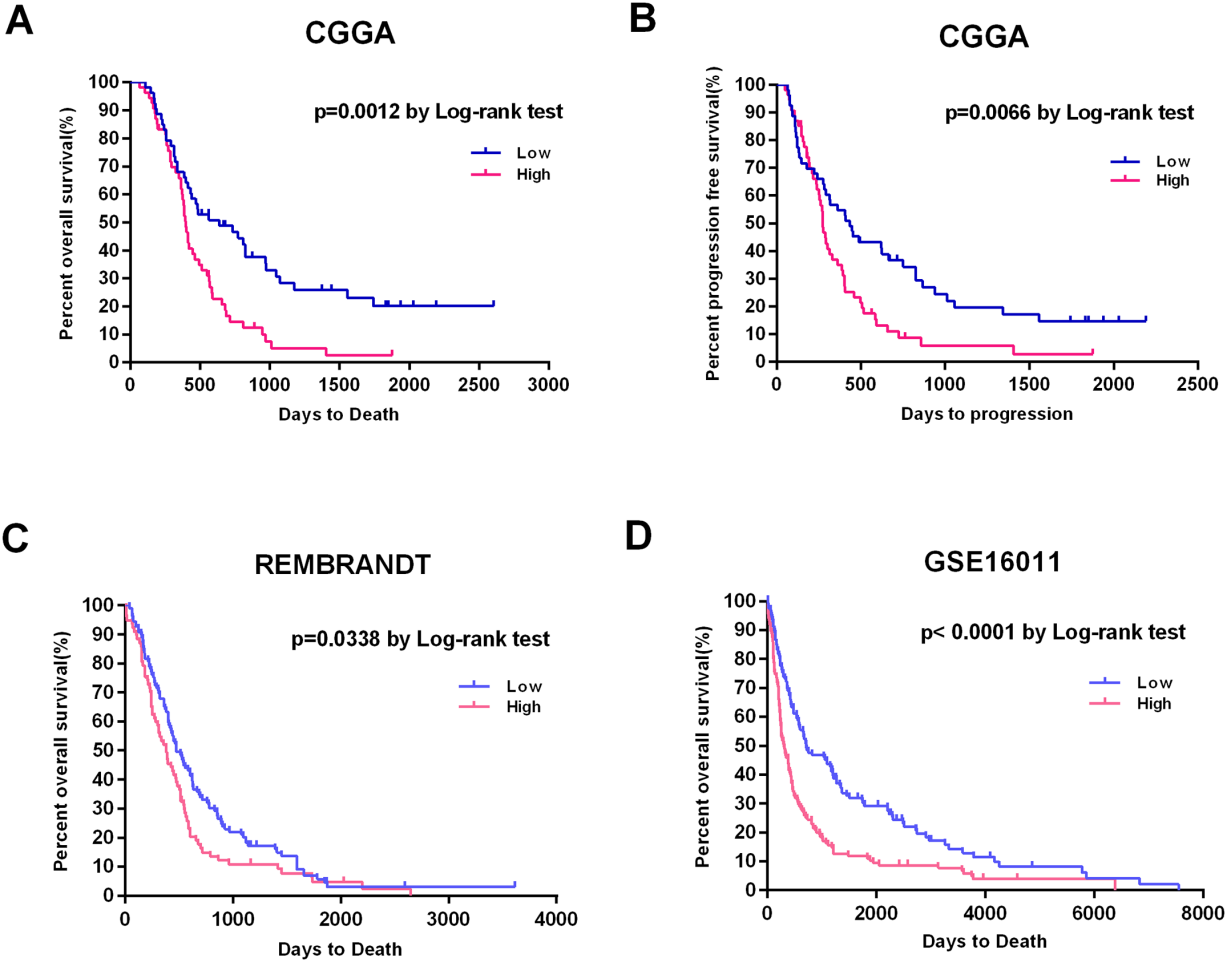
Kaplan-Meier plots of progression-free and overall survival duration in patients with primary GBM. (A, B) Kaplan–Meier survival analysis of PFS and OS duration in 78 primary GBM patients according to *MMP9* mRNA expression. Patients with low *MMP9* expression had a longer OS and PFS than patients with high *MMP9* expression (p = 0.0012 and p = 0.0066, respectively). (C, D) Two independent datasets (REMBRANDT and GSE16011) were used to validate the association between *MMP9* expression and survival. Patients with lower *MMP9* expression also had improved OS in the two validation datasets (p = 0.0338 and p<0.0001, respectively).

We defined the median level of *MMP9* expression of seventy-eight patients with primary GBM as the cutoff value to divide them into low (n = 39) *MMP9* group and high (n = 39) *MMP9* groups (Table 2). The clinicopathologic features of these two groups are shown in Table 2. The patients in the *MMP9* low expression group were younger and had higher rates of *MGMT* promoter methylation and *IDH1* mutation compared to the patients in the *MMP9* high expression group. Patients with low *MMP9* expression had a longer OS and PFS than patients with high *MMP9* expression (p = 0.0012 and p = 0.0066, respectively; Fig 2A and 2B). Then two independent datasets (REMBRANDT and GSE16011) were used to validate the association between *MMP9* expression and survival (Fig 2C and 2D). Consistent with our results, patients with lower *MMP9* expression had improved OS in the two validation datasets (p = 0.0338 and p<0.0001, respectively). Overall, these results indicated that low *MMP9* is expression of *MMP9* correlated with better survival outcome in primary GBMs.

**Table 2:** Clinical and molecular pathological features of primary GBM samples in association with *MMP9* expression.

Total (n = 78)	Low(n = 39)	High(n = 39)	p value
**Gender**			
Male	22	24	0.818
Female	17	15	
**Age at diagnosis**			
≤45	23	9	0.002
>45	16	30	
**Preoperative KPS score**			
≥80	24	19	0.362
<80	15	20	
***MGMT***			
Methylated	20	9	0.017
Unmethylated	18	29	
NA	1	1	
***IDH1***			
Mutation	11	0	<0.001
Wild type	28	39	
**TMZ chemotherapy**			
Yes	28	22	0.157
No	11	17	
**Extent of resection**			
Total	15	12	0.634
Subtotal	24	27	

Abbreviations: *IDH1*, isocitrate dehydrogenase 1; KPS, Karnofsky performance scale; *MGMT*, O6-methylguanine-DNA methyltransferase; TMZ, temozolomide. NA, not available. P values were determined using a 2-sided chi-square test of variance.

We conducted a univariate Cox regression analysis to determine the clinical and genetic variables that were associated with OS for these 78 primary GBM patients (Table 3). *MMP9* expression, preoperative KPS score, age at diagnosis, *MGMT* promoter methylation status and TMZ therapy were statistically associated with OS. We also observed that *MMP9* expression, age at diagnosis and TMZ therapy were statistically associated with PFS. The multivariate Cox regression analysis indicated that *MMP9* expression was an independent prognostic factor for OS and PFS (OS: HR, 1.171; 95% CI, 1.018–1.346; p = 0.027; PFS: HR, 1.146; 95%CI, 1.012–1.299; p = 0.032) (Table 3).

**Table 3:** Cox Hazard Regression Analysis of the Associations of Clinicopathologic Factors and *MMP9* expression for Survival (n = 78).

	Univariate Cox Regression			Multivariate Cox Regression		
Variable	HR	95%CI	p value	HR	95%CI	p value
***Overall survival***						
**Gender**	0.986	0.593–1.640	0.957			
**Age at diagnosis**	1.033	1.011–1.056	0.004	1.011	0.985–1.038	0.404
***MMP9* mRNA expression**	1.248	1.111–1.403	<0.0001	1.171	1.018–1.346	0.027
**Preoperative KPS score**	0.975	0.955–0.995	0.015	0.969	0.948–0.991	0.006
**TMZ chemotherapy**	2.626	1.567–4.401	<0.0001	2.537	1.407–4.575	0.002
***MGMT* promoter methylation**	1.726	0.999–2.979	0.05	1.554	0.861–2.802	0.143
***IDH1* Mutation status**	2.027	0.956–4.295	0.065	1.396	0.590–3.302	0.448
**Extent of surgery**	1.443	0.846–2.462	0.178			
***Progression free survival***						
**Gender**	0.834	0.506–1.377	0.478			
**Age at diagnosis**	1.023	1.002–1.044	0.029	1.004	0.981–1.028	0.71
***MMP9* mRNA expression**	1.2	1.072–1.343	0.002	1.146	1.012–1.299	0.032
**Preoperative KPS score**	0.983	0.964–1.003	0.092			
**TMZ chemotherapy**	2.628	1.579–4.375	<0.0001	2.2	1.280–3.781	0.004
***MGMT* promoter methylation**	1.671	0.986–2.832	0.057			
***IDH1* Mutation status**	1.688	0.829–3.440	0.149			
**Extent of surgery**	1.544	0.914–2.608	0.105			

Abbreviations: KPS, Karnofsky performance status; TMZ: temozolomide; *MGMT*: O6-methylguanine-DNA methyltransferase; *IDH1*: isocitrate dehydrogenase 1; HR: hazard ratio.

Then we also conducted Cox regression analysis to validate the clinical variables and *MMP9* expression in GSE16011 dataset. *MMP9* expression, age at diagnosis were statistically associated with OS (p = 3.08E-9 and p = 6.05E-17, respectively). Because the clinical information of the two datasets was insufficient, we have just used age to adjust in GSE16011 dataset. The multivariate Cox regression analysis indicated that *MMP9* expression was an independent prognostic factor for OS (HR, 2.176; 95% CI, 1.659–2.853; p = 1.94E-8).

### Association between *MMP9* expression and the efficacy of temozolomide chemotherapy

**Fig 3:**
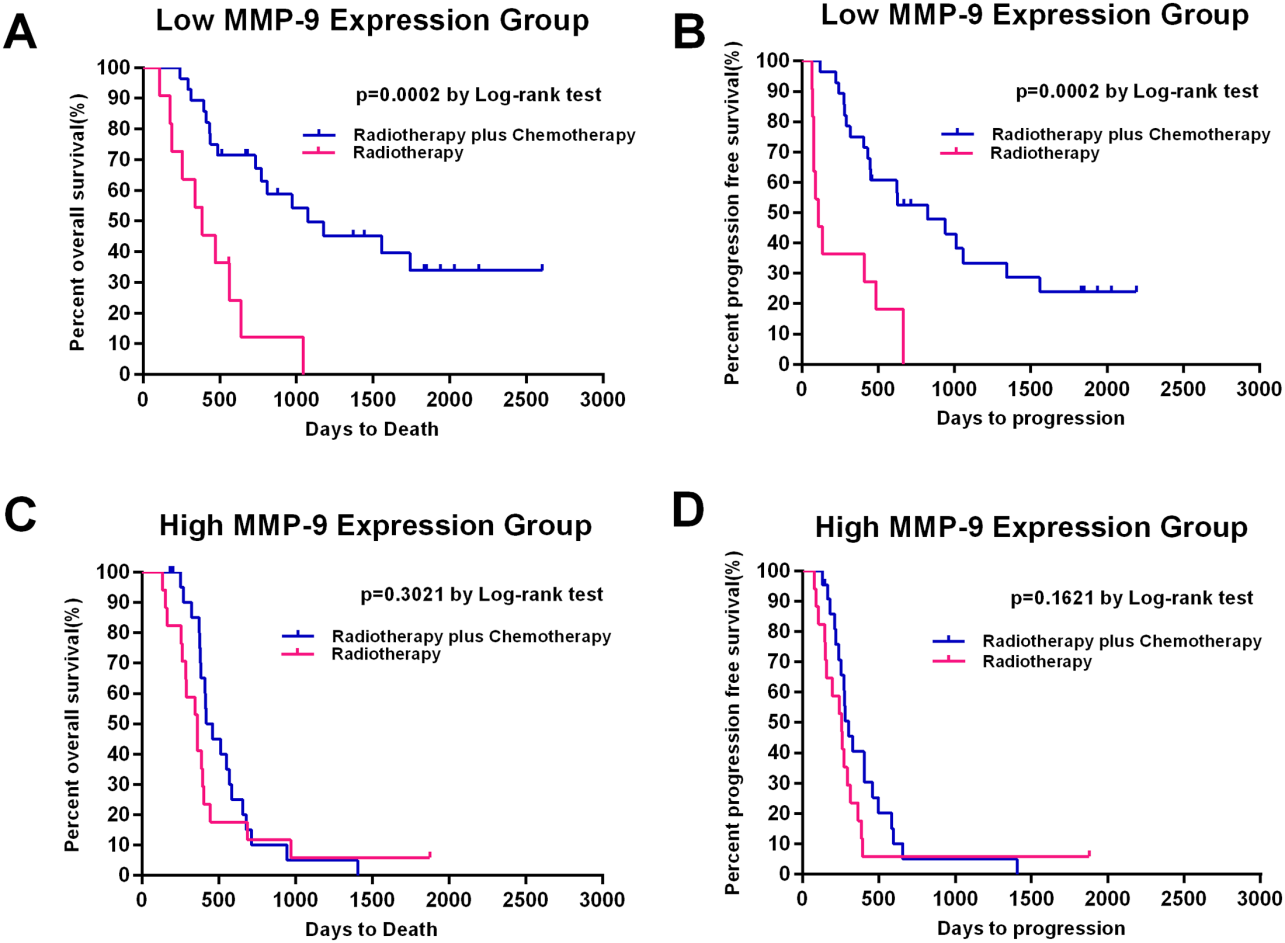
Kaplan-Meier estimates of progression-free and overall survival according to *MMP9* expression and treatment groups. (A, B) Kaplan–Meier survival analysis indicated that patients treated with RT combined with TMZ therapy (n = 28) had better OS and PFS (OS: p = 0.0002; PFS: p = 0.0002) than patients with RT alone (n = 11) in low *MMP9* group (n = 39). (C, D) However, in the high *MMP9* group (n = 39), there was no significant survival benefit of the combination treatment (RT alone: n = 17; RT combined TMZ: n = 22).

To assess the potential association of *MMP9* with the therapeutic outcome of TMZ treatment, we classified the low *MMP9* and high *MMP9* groups into subgroups according to whether TMZ chemotherapy was received. The Kaplan–Meier survival analysis indicated that patients treated with RT combined with TMZ therapy had better OS and PFS (OS: p = 0.0002; PFS: p = 0.0002) than patients treated with RT alone in the low *MMP9* group (Fig 3A and 3B). However, in the high *MMP9* group, there was no significant survival benefit of the combination treatment (Fig 3C and 3D).

**Fig 4:**
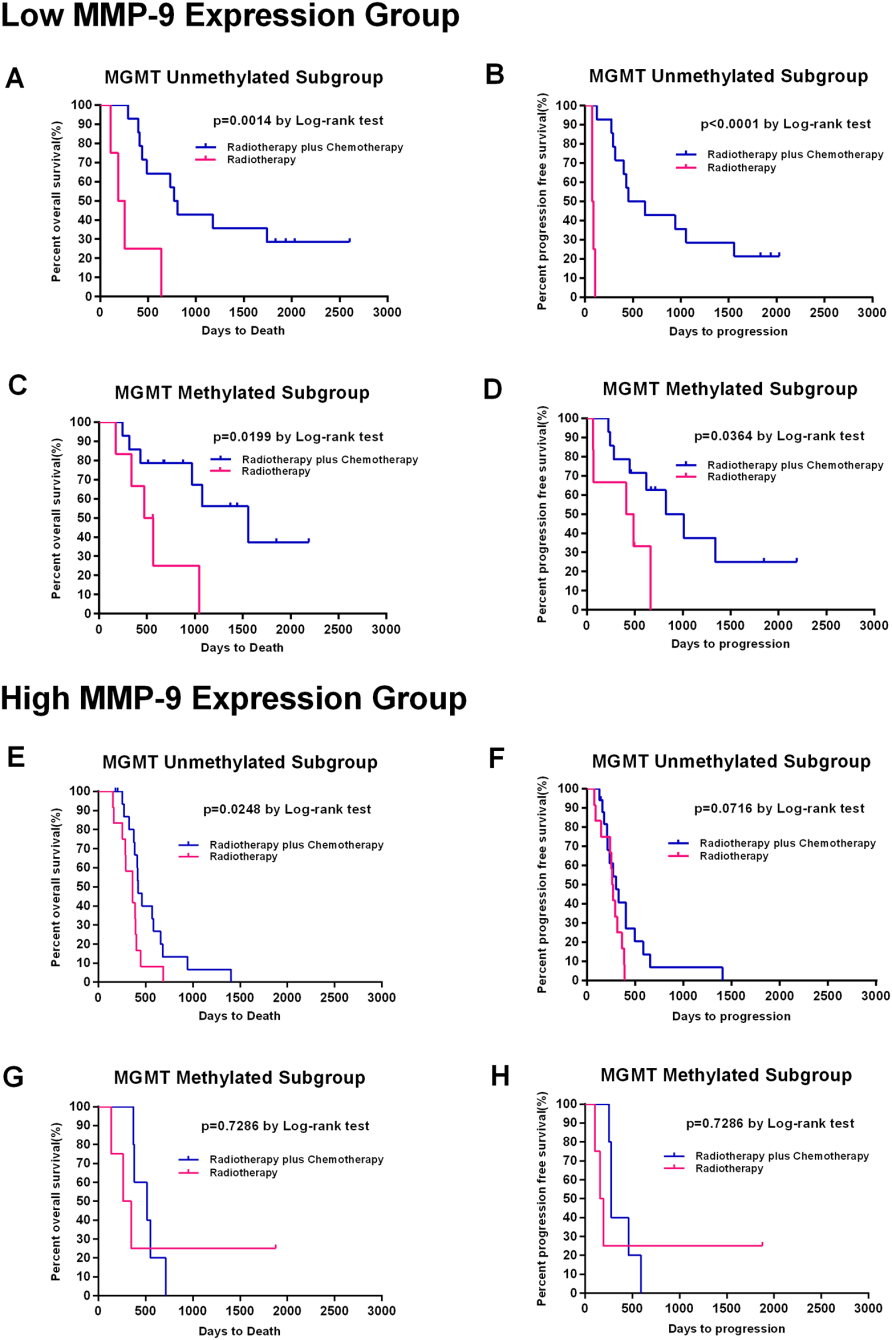
Kaplan-Meier estimates of progression-free and overall survival according to *MMP9* expression, *MGMT* methylation status and treatment groups. (A, B, C, D) In the low *MMP9* group, patients who received the combination therapy showed improved OS and PFS regardless of whether the *MGMT* promoter was methylated (n = 20; RT alone: n = 6; RT combined TMZ: n = 14) or unmethylated (n = 18; RT alone: n = 4; RT combined TMZ: n = 14). (E, F, G, H) In the high *MMP9* group, TMZ chemotherapy resulted in better OS but not better PFS in patients with an unmethylated *MGMT* promoter (n = 29; RT alone: n = 12; RT combined TMZ: n = 17), while TMZ did not benefit patients with a methylated *MGMT* promoter (n = 9; RT alone: n = 4; RT combined TMZ: n = 5).

It is well known that *MGMT* promoter methylation is related to better survival and that patients with a methylated *MGMT* promoter benefit from TMZ chemotherapy[45]. We analyze the correlations of *MGMT* promoter methylation status and *MMP9* expression with TMZ chemotherapy. We divided the low *MMP9* and high *MMP9* groups into subgroups with a methylated and unmethylated *MGMT* promoter. A Kaplan–Meier survival curve analysis with a log-rank comparison was conducted for each subgroup. In the low *MMP9* group, patients who received combined therapy showed improved OS and PFS regardless of *MGMT* methylation status (Fig 4A, 4B, 4C and 4D). In the high *MMP9* group, TMZ chemotherapy resulted in an improved OS but not an improved PFS in patients with an unmethylated *MGMT* promoter (Fig 4E and 4F), while TMZ showed no benefit for patients with a methylated *MGMT* promoter (Fig 4G and 4H). Above results have been validated by cox regression analysis (S4 Table). The CGGA and Rembrandt datasets have been uploaded as S5, S6 and S7 Tables.

## Discussion

Glioblastoma is the most common malignant primary brain tumor in adults. Despite improved surgery and chemo-radiotherapy approaches, the clinical prognosis for patients with GBM remains dismal[2]. The median survival of patients with primary GBM is approximately 1 year, but it varies remarkably from less than few weeks to more than 3 years after diagnosis[46], suggesting the limitations of the current clinicopathologic determinants of prognosis and the choice of therapeutic strategies. Thus, it is of great importance to identify more effective biomarkers that can predict clinical outcomes and therapeutic responses to drugs.

Our paper aimed to identify the prognostic and predictive value of MMPs in patients with primary GBM. Several MMPs have been reported to be related with poor prognosis in a large variety of human cancers [17–19]. In particular, the over-expression of certain MMPs in high-grade gliomas appear to be correlated with tumor invasiveness and to be prognostically significant [47]. MMPs enhance tumor cell invasion by degrading extracellular-matrix proteins, activating signal-transduction cascades that promote motility and solubilizing ECM-bound growth factors[48]. Christopher M. Overall and co-workers introduced the use of proteolytic signature peptides (PSPs) in combination with isobaric tags for the proteomic analysis of MMP proteolytic activity[49]. The association between kep, a perfusion index, and *MMP9* expression has been demonstrated, and kep can be used as an imaging biomarker of GBM progression and its prognostication [50]. In addition, MMPs can cleave and activate other growth factors that are implicated in GBM motility and proliferation, such as TGFβ[51]. In our study, we comparatively analyzed the MMP family members based on whole-gene expression profiling from multiple databases, and confirmed that *MMP9* expression was correlated with glioma grade and that low *MMP9* expression was an independent prognostic factor for better survival in primary GBM patients.

Previous studies have performed experiments to examine the mechanism through which *MMP9* affectes the survival of the glioma patients. *MMP9* is known to play an important role in cell migration and invasion in both physiological and pathological processes of gliomagenesis[52]. Hu et. al. demonstrated that *MMP9* is predominantly expressed by glioma-associated microglia/macrophages in mouse and human glioma tissue not by glioma cells, and glioma-associated microglial *MMP9* expression is upregulated by TLR2 signaling and is sensitive to minocycline[53]. Tie2-expressing monocytes/macrophages are a major source of *MMP9* secretion and activity. After 6 weeks of anti-VEGF therapy, *MMP9* immunostaining of brain tissue sections revealed *MMP9*+ cells at the tumor edge and peripheral invasive tumor nodules with rod or amoeboid shapes characteristic of “activated” microglia/macrophages, and these types of cells were scarcely observed in the control animals[54]. Our team previously used miRNA microarrays to identify the *MMP9*-specific miRNA expression profile of GBM. which may be used to determine potential targets of anti-invasion therapy for GBM [55]. Serum *MMP9* level was determined by ELISA and was found to be correlated with radiographic status and survival [56]. *MMP9* silencing decreased oncogenic c-Myc expression and induced senescence and apoptosis in glioma cells by inhibiting hTERT expression and telomere activity [57]. *MMP9* was also found to be involved in EGFR/Ras/MEK and PI3K/AKT signaling pathway-mediated cell invasion and anchorage-independent growth in U1242 MG cells [58]. Tumor necrosis factor (TNF)-related apoptosis-inducing ligand (TRAIL) induced *MMP9* expression in human astrocytoma cells through activation of extracellular signal-regulated protein kinase (ERK). In addition, TRAIL induced the DNA-binding activity of NF-κB, an important transcription factor for *MMP9* induction[59]. These experiments demonstrated that *MMP9* directly impacts the survival of glioma patients.

Radiation therapy plus TMZ chemotherapy as the first-line treatment for GBM has extended the survival of GBM patients. However, the survival benefit and response to TMZ is variable among patients. The critical reason for the poor prognosis of primary GBM is therapeutic resistance, especially TMZ-resistance, which eventually results in tumor recurrence[60]. It is unclear whether *MMP9* influences the response to TMZ in primary GBM patients. Our data showed an association between *MMP9* expression and the efficacy of temozolomide chemotherapy. TMZ produces the mono-functional DNA adducts O^6^-MeG and N^7^-MeG adducts, and the former is considered a lethal DNA lesion[61]. A report published in *Oncotarget* demonstrated that miR-211 or shRNA-specific for *MMP9* in combination with ionizing radiation and temozolomide significantly induced apoptosis and DNA fragmentation. Additionally, that report showed that glioma stem cells treated with miR-211- and shRNA-specific for *MMP9* (pM) had increased drug retention capacity. [47] These mechanisms may explain why GBM patients with low *MMP9* expression have a better response to TMZ chemotherapy. It is well known that patients with methylation of *MGMT* promoter benefit from TMZ chemotherapy [62]. Therefore, we analyzed the correlations of *MGMT* methylation status and *MMP9* expression with TMZ chemotherapy efficacy. TMZ benefited patients with low *MMP9* expression whether the *MGMT* promoter was methylated or unmethylated. This greatly supports the predictive value of *MMP9* for the response to TMZ. On the other hand, in the high *MMP9* group, TMZ chemotherapy resulted in better OS but not better PFS in patients with an unmethylated *MGMT* promoter, and TMZ did not benefit the patients with a methylated *MGMT* promoter.

In conclusion, we confirmed the association between *MMP9* expression and giloma grade, and highlighted the prognostic and predictive value of *MMP9* among all MMP family members in primary GBMs. These findings suggest that *MMP9* is a potential prognostic and predictive biomarker for glioma and can be used to establish more personalized therapeutic strategies. The US clinical trial “*MMP2*, *MMP9* and *NGAL* as Biomarkers for Glioblastoma (GBM) Biomarkers for the Prognosis of Glioblastoma (NCT01493219)” has been sponsored by University of Nebraska started since 2011. In the future, more work should focus on in-depth molecular mechanisms to provide a more comprehensive understanding of the roles of MMPs in GBM.

## Supporting Information

S1 Table Basic information of the CGGA dataset and the two independent datasets. (DOCX)

S2 Table Multivariate Cox Regression Analysis of the associations between MMPs and survival in the CGGA dataset. (DOCX)

S3 Table Survival analysis of MMPs in the GSE16011 and Rembrandt datasets. (DOCX)

S4 Table Cox Regression Analysis of TMZ chemotherapy for patients with different MGMT methylation and *MMP9* expression. (DOCX)

S5 Table MMPs expression value of the CGGA dataset in our research. (XLSX)

S6 Table MMP9 expression value of the CGGA dataset. (XLSX)

S7 Table MMP9 expression value and follow-up information of the Rembrandt dataset in our research. (XLSX)

## References

[1] Ostrom QT, Gittleman H, Farah P, Ondracek A, Chen Y, Wolinsky Y, et al CBTRUS Statistical Report: Primary Brain and Central Nervous System Tumors Diagnosed in the United States in 2006–2010. Neuro-Oncology. 2013;15(suppl 2):ii1–ii56. 10.1093/neuonc/not151 24137015 PMC3798196

[2] Stupp R, Hegi ME, Mason WP, van den Bent MJ, Taphoorn MJ, Janzer RC, et al Effects of radiotherapy with concomitant and adjuvant temozolomide versus radiotherapy alone on survival in glioblastoma in a randomised phase III study: 5-year analysis of the EORTC-NCIC trial. The lancet oncology. 2009;10(5):459–66. Epub 2009/03/10. 10.1016/s1470-2045(09)70025-7 . 19269895

[3] Liu L, Wu J, Ying Z, Chen B, Han A, Liang Y, et al Astrocyte elevated gene-1 upregulates matrix metalloproteinase-9 and induces human glioma invasion. Cancer Res. 2010;70(9):3750–9. 10.1158/0008-5472.CAN-09-3838 . 20388776

[4] Drappatz J, Norden AD, Wen PY. Therapeutic strategies for inhibiting invasion in glioblastoma. Expert review of neurotherapeutics. 2009;9(4):519–34. 10.1586/ern.09.10 . 19344303

[5] Egeblad M, Werb Z. New functions for the matrix metalloproteinases in cancer progression. Nature reviews Cancer. 2002;2(3):161–74. 10.1038/nrc745 . 11990853

[6] Curran S, Murray GI. Matrix metalloproteinases: molecular aspects of their roles in tumour invasion and metastasis. European journal of cancer. 2000;36(13 Spec No):1621–30. . 10959048 10.1016/s0959-8049(00)00156-8

[7] Liotta LA, Tryggvason K, Garbisa S, Hart I, Foltz CM, Shafie S. Metastatic potential correlates with enzymatic degradation of basement membrane collagen. Nature. 1980;284(5751):67–8. . 6243750 10.1038/284067a0

[8] Nagase H, Woessner JF Jr. Matrix metalloproteinases. The Journal of biological chemistry. 1999;274(31):21491–4. . 10419448 10.1074/jbc.274.31.21491

[9] Nelson AR, Fingleton B, Rothenberg ML, Matrisian LM. Matrix metalloproteinases: biologic activity and clinical implications. Journal of clinical oncology: official journal of the American Society of Clinical Oncology. 2000;18(5):1135–49. . 10694567 10.1200/JCO.2000.18.5.1135

[10] Parsons SL, Watson SA, Brown PD, Collins HM, Steele RJ. Matrix metalloproteinases. The British journal of surgery. 1997;84(2):160–6. . 9052425

[11] Kachra Z, Beaulieu E, Delbecchi L, Mousseau N, Berthelet F, Moumdjian R, et al Expression of matrix metalloproteinases and their inhibitors in human brain tumors. Clinical & experimental metastasis. 1999;17(7):555–66. Epub 2000/06/14. . 10845554 10.1023/a:1006760632766

[12] Stojic J, Hagemann C, Haas S, Herbold C, Kuhnel S, Gerngras S, et al Expression of matrix metalloproteinases MMP-1, MMP-11 and MMP-19 is correlated with the WHO-grading of human malignant gliomas. Neuroscience research. 2008;60(1):40–9. 10.1016/j.neures.2007.09.009 . 17980449

[13] Curran S, Murray GI. Matrix metalloproteinases in tumour invasion and metastasis. The Journal of pathology. 1999;189(3):300–8. 10.1002/(SICI)1096-9896(199911)189:3<300::AID-PATH456>3.0.CO;2-C . 10547590

[14] Hidalgo M, Eckhardt SG. Development of matrix metalloproteinase inhibitors in cancer therapy. Journal of the National Cancer Institute. 2001;93(3):178–93. . 11158186 10.1093/jnci/93.3.178

[15] Roy R, Yang J, Moses MA. Matrix metalloproteinases as novel biomarkers and potential therapeutic targets in human cancer. Journal of clinical oncology: official journal of the American Society of Clinical Oncology. 2009;27(31):5287–97. 10.1200/JCO.2009.23.5556 19738110 PMC2773480

[16] Stetler-Stevenson WG. Type IV collagenases in tumor invasion and metastasis. Cancer metastasis reviews. 1990;9(4):289–303. . 1965794 10.1007/BF00049520

[17] Gao P, Yang JL, Zhao H, You JH, Hu Y. Common polymorphism in the MMP-13 gene may contribute to the risk of human cancers: a meta-analysis. Tumour biology: the journal of the International Society for Oncodevelopmental Biology and Medicine. 2014 10.1007/s13277-014-2309-y . 25023404

[18] Makinen LK, Hayry V, Hagstrom J, Sorsa T, Passador-Santos F, Keski-Santti H, et al Matrix metalloproteinase-7 and matrix metalloproteinase-25 in oral tongue squamous cell carcinoma. Head & neck. 2013 10.1002/hed.23539 . 24488688

[19] Song J, Su H, Zhou YY, Guo LL. Prognostic value of matrix metalloproteinase 9 expression in breast cancer patients: a meta-analysis. Asian Pacific journal of cancer prevention: APJCP. 2013;14(3):1615–21. . 23679245 10.7314/apjcp.2013.14.3.1615

[20] Wang H, Zhang X, Huang L, Li J, Qu S, Pan F. Matrix Metalloproteinase-14 Expression and Its Prognostic Value in Cervical Carcinoma. Cell biochemistry and biophysics. 2014 10.1007/s12013-014-9974-8 . 24789545

[21] Wang J, Li Y, Wang J, Li C, Yu K, Wang Q. Increased expression of matrix metalloproteinase-13 in glioma is associated with poor overall survival of patients. Medical oncology (Northwood, London, England). 2012;29(4):2432–7. 10.1007/s12032-012-0181-4 . 22351249

[22] Yamamoto M, Mohanam S, Sawaya R, Fuller GN, Seiki M, Sato H, et al Differential expression of membrane-type matrix metalloproteinase and its correlation with gelatinase A activation in human malignant brain tumors in vivo and in vitro. Cancer Res. 1996;56(2):384–92. . 8542596

[23] Overall CM, Wrana JL, Sodek J. Transcriptional and post-transcriptional regulation of 72-kDa gelatinase/type IV collagenase by transforming growth factor-beta 1 in human fibroblasts. Comparisons with collagenase and tissue inhibitor of matrix metalloproteinase gene expression. The Journal of biological chemistry. 1991;266(21):14064–71. . 1649834

[24] Kondraganti S, Mohanam S, Chintala SK, Kin Y, Jasti SL, Nirmala C, et al Selective suppression of matrix metalloproteinase-9 in human glioblastoma cells by antisense gene transfer impairs glioblastoma cell invasion. Cancer Res. 2000;60(24):6851–5. . 11156378

[25] Nuttall RK, Pennington CJ, Taplin J, Wheal A, Yong VW, Forsyth PA, et al Elevated membrane-type matrix metalloproteinases in gliomas revealed by profiling proteases and inhibitors in human cancer cells. Molecular cancer research: MCR. 2003;1(5):333–45. . 12651907

[26] Qin H, Moellinger JD, Wells A, Windsor LJ, Sun Y, Benveniste EN. Transcriptional suppression of matrix metalloproteinase-2 gene expression in human astroglioma cells by TNF-alpha and IFN-gamma. Journal of immunology. 1998;161(12):6664–73. . 9862695

[27] Raithatha SA, Muzik H, Muzik H, Rewcastle NB, Johnston RN, Edwards DR, et al Localization of gelatinase-A and gelatinase-B mRNA and protein in human gliomas. Neuro Oncol. 2000;2(3):145–50. 11302334 10.1093/neuonc/2.3.145 PMC1920497

[28] Hagemann C, Anacker J, Ernestus RI, Vince GH. A complete compilation of matrix metalloproteinase expression in human malignant gliomas. World journal of clinical oncology. 2012;3(5):67–79. Epub 2012/05/15. 10.5306/wjco.v3.i5.67 ; PubMed Central PMCID: PMCPmc3349915. 22582165 PMC3349915

[29] Llano E, Pendas AM, Freije JP, Nakano A, Knauper V, Murphy G, et al Identification and characterization of human MT5-MMP, a new membrane-bound activator of progelatinase a overexpressed in brain tumors. Cancer Res. 1999;59(11):2570–6. . 10363975

[30] Lampert K, Machein U, Machein MR, Conca W, Peter HH, Volk B. Expression of matrix metalloproteinases and their tissue inhibitors in human brain tumors. The American journal of pathology. 1998;153(2):429–37. Epub 1998/08/26. 10.1016/s0002-9440(10)65586-1 ; PubMed Central PMCID: PMCPmc1852969. 9708803 PMC1852969

[31] Cai J, Chen J, Zhang W, Yang P, Zhang C, Li M, et al Loss of ATRX, associated with DNA methylation pattern of chromosome end, impacted biological behaviors of astrocytic tumors. Oncotarget. 2015. 10.18632/oncotarget.3906 PMC4627238 25971279

[32] Louis DN, Ohgaki H, Wiestler OD, Cavenee WK, Burger PC, Jouvet A, et al The 2007 WHO classification of tumours of the central nervous system. Acta Neuropathol. 2007;114(2):97–109. Epub 2007/07/10. 10.1007/s00401-007-0243-4 ; PubMed Central PMCID: PMCPmc1929165. 17618441 PMC1929165

[33] Gravendeel LA, Kouwenhoven MC, Gevaert O, de Rooi JJ, Stubbs AP, Duijm JE, et al Intrinsic gene expression profiles of gliomas are a better predictor of survival than histology. Cancer Res. 2009;69(23):9065–72. 10.1158/0008-5472.CAN-09-2307 . 19920198

[34] Madhavan S, Zenklusen JC, Kotliarov Y, Sahni H, Fine HA, Buetow K. Rembrandt: helping personalized medicine become a reality through integrative translational research. Molecular cancer research: MCR. 2009;7(2):157–67. 10.1158/1541-7786.MCR-08-0435 19208739 PMC2645472

[35] Yan W, Zhang W, You G, Zhang J, Han L, Bao Z, et al Molecular classification of gliomas based on whole genome gene expression: a systematic report of 225 samples from the Chinese Glioma Cooperative Group. Neuro Oncol. 2012;14(12):1432–40. 10.1093/neuonc/nos263 23090983 PMC3499016

[36] Liu Y, Hu H, Wang K, Zhang C, Wang Y, Yao K, et al Multidimensional analysis of gene expression reveals TGFB1I1-induced EMT contributes to malignant progression of astrocytomas. Oncotarget. 2014 . 25333259 10.18632/oncotarget.2518 PMC4350345

[37] Zhang JX, Han L, Bao ZS, Wang YY, Chen LY, Yan W, et al HOTAIR, a cell cycle-associated long noncoding RNA and a strong predictor of survival, is preferentially expressed in classical and mesenchymal glioma. Neuro Oncol. 2013;15(12):1595–603. 10.1093/neuonc/not131 24203894 PMC3829598

[38] Bao ZS, Li MY, Wang JY, Zhang CB, Wang HJ, Yan W, et al Prognostic Value of a Nine-Gene Signature in Glioma Patients Based on mRNA Expression Profiling. CNS Neurosci Ther. 2013 10.1111/cns.12171 . 24279471 PMC6493176

[39] Bao ZS, Zhang CB, Wang HJ, Yan W, Liu YW, Li MY, et al Whole-genome mRNA expression profiling identifies functional and prognostic signatures in patients with mesenchymal glioblastoma multiforme. CNS Neurosci Ther. 2013;19(9):714–20. 10.1111/cns.12118 . 23663361 PMC6493663

[40] Kitchen RR, Sabine VS, Simen AA, Dixon JM, Bartlett JM, Sims AH. Relative impact of key sources of systematic noise in Affymetrix and Illumina gene-expression microarray experiments. BMC genomics. 2011;12:589 10.1186/1471-2164-12-589 22133085 PMC3269440

[41] Kitchen RR, Sabine VS, Sims AH, Macaskill EJ, Renshaw L, Thomas JS, et al Correcting for intra-experiment variation in Illumina BeadChip data is necessary to generate robust gene-expression profiles. BMC genomics. 2010;11:134 10.1186/1471-2164-11-134 20181233 PMC2843619

[42] Walker WL, Liao IH, Gilbert DL, Wong B, Pollard KS, McCulloch CE, et al Empirical Bayes accomodation of batch-effects in microarray data using identical replicate reference samples: application to RNA expression profiling of blood from Duchenne muscular dystrophy patients. BMC genomics. 2008;9:494 10.1186/1471-2164-9-494 18937867 PMC2576259

[43] Cai J, Yang P, Zhang C, Zhang W, Liu Y, Bao Z, et al ATRX mRNA expression combined with IDH1/2 mutational status and Ki-67 expression refines the molecular classification of astrocytic tumors: evidence from the whole transcriptome sequencing of 169 samples samples. Oncotarget. 2014 . 24810474 10.18632/oncotarget.1838 PMC4058026

[44] Cai J, Zhang W, Yang P, Wang Y, Li M, Zhang C, et al Identification of a 6-Cytokine Prognostic Signature in Patients with Primary Glioblastoma Harboring M2 Microglia/Macrophage Phenotype Relevance. PLoS One. 2015;10(5):e0126022 10.1371/journal.pone.0126022 . 25978454 PMC4433225

[45] Hegi ME, Diserens AC, Gorlia T, Hamou MF, de Tribolet N, Weller M, et al MGMT gene silencing and benefit from temozolomide in glioblastoma. The New England journal of medicine. 2005;352(10):997–1003. 10.1056/NEJMoa043331 . 15758010

[46] Ohgaki H, Dessen P, Jourde B, Horstmann S, Nishikawa T, Di Patre PL, et al Genetic pathways to glioblastoma: a population-based study. Cancer Res. 2004;64(19):6892–9. 10.1158/0008-5472.CAN-04-1337 . 15466178

[47] Asuthkar S, Velpula KK, Chetty C, Gorantla B, Rao JS. Epigenetic regulation of miRNA-211 by MMP-9 governs glioma cell apoptosis, chemosensitivity and radiosensitivity. Oncotarget. 2012;3(11):1439–54. 23183822 10.18632/oncotarget.683 PMC3717804

[48] McCawley LJ, Matrisian LM. Matrix metalloproteinases: they're not just for matrix anymore! Current opinion in cell biology. 2001;13(5):534–40. . 11544020 10.1016/s0955-0674(00)00248-9

[49] Fahlman RP, Chen W, Overall CM. Absolute proteomic quantification of the activity state of proteases and proteolytic cleavages using proteolytic signature peptides and isobaric tags. Journal of proteomics. 2014;100:79–91. 10.1016/j.jprot.2013.09.006 . 24060996

[50] Awasthi R, Pandey CM, Sahoo P, Behari S, Kumar V, Kumar S, et al Dynamic contrast-enhanced magnetic resonance imaging-derived kep as a potential biomarker of matrix metalloproteinase 9 expression in patients with glioblastoma multiforme: a pilot study. Journal of computer assisted tomography. 2012;36(1):125–30. 10.1097/RCT.0b013e31823f6c59 . 22261782

[51] Platten M, Wick W, Weller M. Malignant glioma biology: role for TGF-beta in growth, motility, angiogenesis, and immune escape. Microscopy research and technique. 2001;52(4):401–10. 10.1002/1097-0029(20010215)52:4<401::AID-JEMT1025>3.0.CO;2-C . 11170299

[52] Fisher JF, Mobashery S. Mechanism-based profiling of MMPs. Methods in molecular biology. 2010;622:471–87. 10.1007/978-1-60327-299-5_27 . 20135299 PMC6986384

[53] Hu F, Ku MC, Markovic D, a Dzaye OD, Lehnardt S, Synowitz M, et al Glioma-associated microglial MMP9 expression is upregulated by TLR2 signaling and sensitive to minocycline. International journal of cancer Journal international du cancer. 2014;135(11):2569–78. Epub 2014/04/23. 10.1002/ijc.28908 . 24752463 PMC4519695

[54] Gabrusiewicz K, Liu D, Cortes-Santiago N, Hossain MB, Conrad CA, Aldape KD, et al Anti-vascular endothelial growth factor therapy-induced glioma invasion is associated with accumulation of Tie2-expressing monocytes. Oncotarget. 2014;5(8):2208–20. 24809734 10.18632/oncotarget.1893 PMC4039157

[55] Yan W, Zhang W, Sun L, Liu Y, You G, Wang Y, et al Identification of MMP-9 specific microRNA expression profile as potential targets of anti-invasion therapy in glioblastoma multiforme. Brain Res. 2011;1411:108–15. 10.1016/j.brainres.2011.07.002 . 21831363

[56] Hormigo A, Gu B, Karimi S, Riedel E, Panageas KS, Edgar MA, et al YKL-40 and matrix metalloproteinase-9 as potential serum biomarkers for patients with high-grade gliomas. Clinical cancer research: an official journal of the American Association for Cancer Research. 2006;12(19):5698–704. 10.1158/1078-0432.CCR-06-0181 . 17020973

[57] Ponnala S, Chetty C, Veeravalli KK, Dinh DH, Klopfenstein JD, Rao JS. MMP-9 silencing regulates hTERT expression via beta1 integrin-mediated FAK signaling and induces senescence in glioma xenograft cells. Cellular signalling. 2011;23(12):2065–75. 10.1016/j.cellsig.2011.08.001 21855630 PMC3184383

[58] Zhao Y, Xiao A, diPierro CG, Carpenter JE, Abdel-Fattah R, Redpath GT, et al An extensive invasive intracranial human glioblastoma xenograft model: role of high level matrix metalloproteinase 9. The American journal of pathology. 2010;176(6):3032–49. 10.2353/ajpath.2010.090571 20413683 PMC2877863

[59] Kim JH, Choi C, Benveniste EN, Kwon D. TRAIL induces MMP-9 expression via ERK activation in human astrocytoma cells. Biochem Biophys Res Commun. 2008;377(1):195–9. 10.1016/j.bbrc.2008.09.095 . 18834856

[60] Hou LC, Veeravagu A, Hsu AR, Tse VC. Recurrent glioblastoma multiforme: a review of natural history and management options. Neurosurgical focus. 2006;20(4):E5 . 16709036 10.3171/foc.2006.20.4.2

[61] Liu L, Markowitz S, Gerson SL. Mismatch repair mutations override alkyltransferase in conferring resistance to temozolomide but not to 1,3-bis(2-chloroethyl)nitrosourea. Cancer Res. 1996;56(23):5375–9. . 8968088

[62] Stupp R, Mason WP, van den Bent MJ, Weller M, Fisher B, Taphoorn MJ, et al Radiotherapy plus concomitant and adjuvant temozolomide for glioblastoma. The New England journal of medicine. 2005;352(10):987–96. 10.1056/NEJMoa043330 . 15758009

