# Supplementary material for: Comparative Analysis of Matrix Metalloproteinase Family Members Reveals That MMP9 Predicts Survival and Response to Temozolomide in Patients with Primary Glioblastoma
Source: PLoS One. 2016 Mar 29;11(3):e0151815. doi: 10.1371/journal.pone.0151815 (PMC4811585; doi:10.1371/journal.pone.0151815)
Supplement: S1 Table — (DOCX) [file pone.0151815.s001.docx]

**Supplementary Table S1. Basic information of the CGGA dataset and the two independent datasets.**

|  |  | CGGA | REMBRANDT | GSE16011 |
| --- | --- | --- | --- | --- |
| *Total* |  | 305 | 433 | 272 |
| *Age* | *≤45* | 184 |  | 106 |
|  | *＞45* | 121 |  | 166 |
| *Gender* | *Male* | 181 | 220 | 182 |
|  | *Female* | 124 | 125 | 90 |
|  | *NA* |  | 88 |  |
| *Grade* | *II* | 126 | 99 | 24 |
|  | *III* | 51 | 84 | 85 |
|  | *IV* | 128 | 211 | 159 |
|  | *NA* |  | 39 | 4 |
| *Histology* | *Astrocytoma* | 84 | 148 | 29 |
|  | *Oligodendroglioma* | 41 | 63 | 52 |
|  | *Oligoastrocytoma* | 52 | 11 | 28 |
|  | *Glioblastoma* | 128 | 211 | 159 |
|  | *NA* |  |  | 4 |
| *TMZ* | *YES* | 141 |  |  |
|  | *NO* | 132 |  |  |
|  | *NA* | 32 |  |  |
