# Supplementary material for: Comparative Analysis of Matrix Metalloproteinase Family Members Reveals That MMP9 Predicts Survival and Response to Temozolomide in Patients with Primary Glioblastoma
Source: PLoS One. 2016 Mar 29;11(3):e0151815. doi: 10.1371/journal.pone.0151815 (PMC4811585; doi:10.1371/journal.pone.0151815)
Supplement: S2 Table — (DOCX) [file pone.0151815.s002.docx]

| Supplementary Table S2. Multivariate Cox Regression Analysis of MMPs for survival in CGGA dataset. | | | |
| --- | --- | --- | --- |
| MMPs | **HR** | **95%CI** | **p value** |
| *MMP9* | 1.395 | 1.144-1.701 | 0.001 |
| *MMP1* | 1.078 | 0.841-1.383 | 0.552 |
| *MMP19* | 0.907 | 0.617-1.334 | 0.621 |
| *MMP7* | 1.042 | 0.896-1.213 | 0.59 |
| *MMP28* | 0.669 | 0.417-1.073 | 0.096 |
| *MMP11* | 0.76 | 0.591-0.977 | 0.033 |
|  | | | |
