# Supplementary material for: Comparative Analysis of Matrix Metalloproteinase Family Members Reveals That MMP9 Predicts Survival and Response to Temozolomide in Patients with Primary Glioblastoma
Source: PLoS One. 2016 Mar 29;11(3):e0151815. doi: 10.1371/journal.pone.0151815 (PMC4811585; doi:10.1371/journal.pone.0151815)
Supplement: S3 Table — (DOCX) [file pone.0151815.s003.docx]

| **Supplementary Table S3. Survival analysis of MMPs Families in GSE16011 and Rembrandt datasets.** | | | | | | | | | |  |
| --- | --- | --- | --- | --- | --- | --- | --- | --- | --- | --- |
|  |  | **GSE16011** | |  |  |  | **Rembrandt** | |  | |
| **MMPs** | **HR** | **95% CI** | | **p** |  | **HR** | **95% CI** | | **p** | |
| ***MMP1*** | 1.0763 | 0.9570 | 1.2104 | 0.2201 |  | 1.0310 | 0.8671 | 1.2260 | 0.7296 | |
| ***MMP2*** | 1.4968 | 0.8846 | 2.5328 | 0.1328 |  | 1.6028 | 0.8969 | 2.8643 | 0.1113 | |
| ***MMP3*** | 0.8962 | 0.6903 | 1.1636 | 0.4107 |  | 1.8080 | 0.9480 | 3.4480 | 0.0722 | |
| ***MMP7*** | 1.0397 | 0.9275 | 1.1655 | 0.5035 |  | 1.1023 | 0.9738 | 1.2478 | 0.1236 | |
| ***MMP8*** | 2.3085 | 0.9854 | 5.4084 | 0.0541 |  | 1.0856 | 0.5436 | 2.1681 | 0.8159 | |
| ***MMP9*** | 1.1149 | 1.0056 | 1.2360 | 0.0389 |  | 1.1099 | 1.0009 | 1.2306 | 0.0480 | |
| ***MMP10*** | 1.0402 | 0.7013 | 1.5431 | 0.8445 |  | 0.7842 | 0.3898 | 1.5778 | 0.4955 | |
| ***MMP11*** | 1.4168 | 0.7957 | 2.5225 | 0.2366 |  | 1.3064 | 0.6098 | 2.7988 | 0.4918 | |
| ***MMP12*** | 0.9899 | 0.8934 | 1.0968 | 0.8461 |  | 0.9398 | 0.7648 | 1.1549 | 0.5552 | |
| ***MMP13*** | 1.1184 | 0.9297 | 1.3453 | 0.2353 |  | 1.0775 | 0.5927 | 1.9588 | 0.8066 | |
| ***MMP14*** | 1.1115 | 0.9042 | 1.3662 | 0.3155 |  | 1.5020 | 1.1627 | 1.9403 | 0.0018 | |
| ***MMP15*** | 0.8160 | 0.4672 | 1.4253 | 0.4749 |  | 1.5258 | 0.7636 | 3.0488 | 0.2315 | |
| ***MMP16*** | 0.7966 | 0.6611 | 0.9598 | 0.0168 |  | 0.9971 | 0.8120 | 1.2244 | 0.9781 | |
| ***MMP17*** | 0.8458 | 0.5874 | 1.2178 | 0.3678 |  | 0.9795 | 0.6814 | 1.4081 | 0.9110 | |
| ***MMP19*** | 1.3284 | 1.0384 | 1.6995 | 0.0238 |  | 1.3495 | 0.9337 | 1.9504 | 0.1108 | |
| ***MMP20*** | 2.3170 | 0.7231 | 7.4243 | 0.1573 |  | 0.3164 | 0.1108 | 0.9031 | 0.0315 | |
| ***MMP21*** | 0.3407 | 0.1096 | 1.0590 | 0.0628 |  | 0.1080 | 0.0225 | 0.5192 | 0.0055 | |
| ***MMP24*** | 0.7426 | 0.4566 | 1.2077 | 0.2303 |  | 0.6110 | 0.3332 | 1.1203 | 0.1112 | |
| ***MMP25*** | 0.5710 | 0.2776 | 1.1745 | 0.1278 |  | 0.8093 | 0.3794 | 1.7263 | 0.5840 | |
| ***MMP26*** | 1.5329 | 0.5367 | 4.3784 | 0.4250 |  | 0.5774 | 0.2652 | 1.2570 | 0.1664 | |
| ***MMP27*** | 1.0036 | 0.4021 | 2.5054 | 0.9938 |  | 0.3651 | 0.1336 | 0.9977 | 0.0495 | |
| ***MMP28*** | 1.2396 | 0.8799 | 1.7463 | 0.2194 |  | 0.7697 | 0.5159 | 1.1483 | 0.1996 | |
