# Supplementary material for: Comparative Analysis of Matrix Metalloproteinase Family Members Reveals That MMP9 Predicts Survival and Response to Temozolomide in Patients with Primary Glioblastoma
Source: PLoS One. 2016 Mar 29;11(3):e0151815. doi: 10.1371/journal.pone.0151815 (PMC4811585; doi:10.1371/journal.pone.0151815)
Supplement: S4 Table — (DOCX) [file pone.0151815.s004.docx]

**Supplementary Table S4. Cox Regression Analysis of TMZ chemotherapy for patients with different MGMT methylation and MMP-9 expression.**

| Variable | HR | 95%CI | p value |
| --- | --- | --- | --- |
| *Low MMP-9 expression group* |  |  |  |
| *Overall survival* |  |  |  |
| MGMT unmethylated subgroup | 6.815 | 1.771-26.232 | 0.005 |
| MGMT methylated subgroup | 4.344 | 1.137-16.604 | 0.032 |
| *Progression free survival* |  |  |  |
| MGMT unmethylated subgroup | 8.152 | 1.899-32.479 | 0.003 |
| MGMT methylated subgroup | 3.549 | 1.004-12.543 | 0.049 |
| *High MMP-9 expression group* |  |  |  |
| *Overall survival* |  |  |  |
| MGMT unmethylated subgroup | 2.319 | 1.034-5.202 | 0.041 |
| MGMT methylated subgroup | 1.298 | 0.297-5.674 | 0.729 |
| *Progression free survival* |  |  |  |
| MGMT unmethylated subgroup | 2.201 | 0.914-5.303 | 0.079 |
| MGMT methylated subgroup | 1.298 | 0.297-5.674 | 0.729 |
